# Case–control study of risk factors for infectious mastitis in Spanish breastfeeding women

**DOI:** 10.1186/1471-2393-14-195

**Published:** 2014-06-06

**Authors:** Pilar Mediano, Leónides Fernández, Juan M Rodríguez, María Marín

**Affiliations:** 1 Departamento de Nutrición, Bromatología y Tecnología de los Alimentos, Universidad Complutense de Madrid, 28040 Madrid, Spain

**Keywords:** Breastfeeding, Infectious mastitis, Risk factors, Public health, Epidemiology

## Abstract

**Background:**

The purpose of this study was to identify potential predisposing factors associated with human infectious mastitis.

**Methods:**

We conducted a case–control study among breastfeeding women, with 368 cases (women with mastitis) and 148 controls. Data were collected by a questionnaire designed to obtain retrospective information about several factors related to medical history of mother and infant, different aspects of pregnancy, delivery and postpartum, and breastfeeding practices that could be involved in mastitis. Bivariate analyses and multivariate logistic regression model were used to examine the relationship between mastitis and these factors.

**Results:**

The variables significantly- and independently-associated with mastitis were cracked nipples (*P* < 0.0001), oral antibiotics during breastfeeding (*P* < 0.0001), breast pumps (*P* < 0.0001), topical antifungal medication during breastfeeding (*P* = 0.0009), mastitis in previous lactations (*P* = 0.0014), breast milk coming in later than 24 h postpartum (*P* = 0.0016), history of mastitis in the family (*P* = 0.0028), mother-infant separation longer than 24 h (*P* = 0.0027), cream on nipples (*P* = 0.0228) and throat infection (*P* = 0.0224).

**Conclusions:**

Valuable factors related to an increased risk of infectious mastitis have been identified. This knowledge will allow practitioners to provide appropriate management advice about modifiable risk factors, such as the use of pumps or inappropriate medication. They also could identify before delivery those women at an increased risk of developing mastitis, such as those having a familial history of mastitis, and thus develop strategies to prevent this condition.

## Background

Infectious mastitis is a common condition that affects up to 33% of women during lactation, although its incidence may be underestimated because of differences in case definition and reporting [1-4]. However, as it has recently been addressed [5], most of the studies have not reported the true mastitis incidence to date, since it would be necessary to define a time limit for the collection of data and to know the size of the population at risk, that is, breastfeeding mothers in the area of study. On one hand, the term “infectious mastitis” has been applied only to acute cases, with both local (breast redness, engorgement and pain) and systemic symptoms; however, subacute mastitis, that include only local symptoms and are often characterized by a reduced milk secretion, have been systematically underreported. On the other hand, human milk cultures are rarely performed and, therefore, there are not standardized sampling and analysis procedures [6].

Lactational mastitis constitutes one of the main medical causes of premature weaning due to pain and discomfort or as a result of inappropriate advise of a health professional [7,8]. Since breastfeeding provides a wide range of health benefits for the mother-infant pair [9-11], mastitis constitutes a relevant Public Health issue [6,12]. Some epidemiologic studies have been carried out to investigate the incidence and the potential risk factors that could be involved in infectious lactational mastitis [1,3,7,13-15]. Risk factors that have been suggested to be strongly associated to mastitis include, among others, mastitis with a previous child, cracked or sore nipples, use of ointments, inappropriate breastfeeding practices, and peripartum antibiotherapy [1,4,13-15].

In Spain, the most recent National Survey of Health available (2011–2012) showed that the estimated prevalence of exclusive (mixed) breastfeeding was 66.2 (72.4)%, 53.6 (66.6)% and 28.5 (46.9)% at 6 weeks, 3 months and 6 months, respectively, after birth [16]. On the other hand, Spain ranks regular (24 out of 36) in the *Breastfeeding Policy Scorecard for Developed Countries* published recently [17] according to maternity leave laws and right to daily nursing breaks, among other indicators. Furthermore, the number of centers that hold the *Baby Friendly Hospital* designation as institutions that promote breastfeeding, as well as the *Breastfeeding Support Groups*, has increased during the last decade. However, in contrast to this renewed interest that breastfeeding and human milk are receiving nowadays, lactational mastitis still remains widely unknown to the medical community, including the Spanish practitioners. In this sense, the *Recommendations on Breastfeeding of the Lactation Committee of the Spanish Association of Paediatrics*[18] do not even mention mastitis as a common condition during lactation and one of the main medical causes of premature weaning.

In this study, a broad range of risk factors have been evaluated in a Spanish population. We have addressed some risks factors from literature and others that have not been taken in account in previous studies. To our knowledge this is the first large epidemiological study about risk factors for infectious mastitis among lactating women in a Spanish population.

## Methods

### Subject selection

Cases (n = 368) who participated in this retrospective case–control study by filling out a questionnaire about mastitis risk factors, were recruited from 1080 lactating women with clinical symptoms of infectious mastitis who attended our laboratory from September 2009 to June 2011 to have a breast milk sample analyzed in the context of a study about microbiology of human mastitis.

They were referred to our laboratory by lactation consultants and midwives attending different health-care centers in Spain and all cases included either both local (breast redness, pain and engorgement) and systemic symptoms (fever or flu-like symptoms) or only local symptoms (pain, engorgement, reduced milk secretion). Patients suffering from Raynaud’s syndrome, mammary abscesses or any other mammary pathology were excluded from the study. The diagnosis of mastitis was confirmed by milk cultures that were plated onto ready-to-use Baird Parker (selective medium for staphylococci isolation) and Columbia Blood Agar for isolation of streptococci, staphylococci, corynebacteria and related bacteria. Violet Red Bile Glucose Agar was used for isolation of enterobacteria and other Gram-negative bacteria in order to confirm that the milk samples had not been contaminated. The plates were incubated at 37°C for 48 h and to reach a positive diagnosis of mastitis the following microbiological criteria was established: *Staphylococcus aureus* > 150 CFU/mL, coagulase-negative staphylococci (mainly *Staphylococcus epidermidis*) > 1000 CFU/mL and viridans streptococci (mainly *Streptococcus mitis* and *Streptococcus salivarius*) > 1000 CFU/mL [19].

Midwives and lactation consultants were also asked to recruit healthy breastfeeding women with no clinical symptoms of mastitis during current lactation as the control group. In order to verify the absence of mastitis in the controls, breast milk samples were also cultured in the same media as mastitis samples. The mammary microbiota in controls was characterized by the presence of a relatively heterogeneous population at a moderated concentration (<1000 CFU/mL) and < 10^3^ white blood cells per mL of milk. Informed consent to the protocol approved by the Ethical Committee of Hospital Clínico San Carlos (Madrid, Spain) was obtained from the women involved in this study.

Cases and controls (all of them with full-term pregnancy and healthy children) were asked to fill out a questionnaire with precoded and open-ended questions designed to collect retrospective information on demographic characteristics, medical history of mother and infant, different aspects of pregnancy, delivery and postpartum, and breastfeeding practices (Tables 1, 2, 3 and 4). The questionnaire was completed and returned by 368 out of 1080 cases (34%) and 148 out of 256 controls (58%).

**Table 1:** Medical history of women participating in the study

**Variables**	**Case, *n*** * (%)	**Control, *n*** * (%)	**OR**	**95% CI**	***P*-value**
**Blood type**					
A	158 (44.13)	74 (52.11)	Reference		0.369
B	28 (7.82)	12 (8.45)	1.09	0.53–2.27	
AB	19 (5.31)	5 (3.52)	1.78	0.64–4.95	
O	153 (42.74)	51 (35.92)	1.41	0.92–2.14	
**Rh factor**					
Positive	275 (78.13)	121 (85.82)	Reference		0.052
Negative	77 (21.88)	20 (14.18)	1.69	0.99–2.90	
**Mastitis in the family**					
No	191 (62.01)	120 (83.92)	Reference		< 0.001
Yes	117 (37.99)	23 (16.08)	3.20	1.93–5.28	
Mastitis in previous breastfeedings**					
No	53 (48.62)	54 (87.10)	Reference		< 0.001
Yes	56 (51.38)	8 (12.90)	7.13	3.10–16.39	
**Breast cancer in the family**					
No	265 (74.44)	99 (68.28)	Reference		0.161
Yes	91 (25.56)	46 (31.72)	0.74	0.48–1.13	
**Breast surgery**					
No	341 (94.99)	141 (97.24)	Reference		0.378
Yes	18 (5.01)	4 (2.76)	1.86	0.62–5.60	
**Gastrointestinal disease**					
No	267 (74.17)	113 (76.87)	Reference		0.524
Yes	93 (25.83)	34 (23.13)	1.16	0.74–1.82	
**Urinary infection**					
No	286 (78.36)	128 (86.49)	Reference		0.035
Yes	79 (21.64)	20 (13.51)	1.77	1.04–3.01	
**Vaginal candidiasis**					
No	279 (76.23)	127 (85.81)	Reference		0.016
Yes	87 (23.77)	21 (14.19)	1.89	1.12–3.17	
**Eye infection**					
No	335 (91.78)	139 (93.92)	Reference		0.408
Yes	30 (8.22)	9 (6.08)	1.38	0.64–2.99	
**Ear infection**					
No	350 (95.89)	145 (97.97)	Reference		0.370
Yes	15 (4.11)	3 (2.03)	2.07	0.59–7.26	
**Lip or nose infection**					
No	285 (77.87)	125 (84.46)	Reference		0.092
Yes	81 (22.13)	23 (15.54)	1.54	0.93–2.57	
**Throat infection**					
No	258 (70.88)	126 (85.71)	Reference		< 0.001
Yes	106 (29.12)	21 (14.29)	2.47	1.47–4.12	
**Skin infection**					
No	301 (82.47)	137 (92.57)	Reference		0.003
Yes	64 (17.53)	11 (7.43)	2.65	1.35–5.18	
**Allergies**					
No	250 (68.31)	106 (71.62)	Reference		0.461
Yes	116 (31.69)	42 (28.38)	1.17	0.77–1.78	
**Autoimmune disease**					
No	352 (96.17)	146 (98.65)	Reference		0.237
Yes	14 (3.83)	2 (1.35)	2.90	0.65–12.94	
**Asthma**					
No	347 (94.81)	140 (94.59)	Reference		0.922
Yes	19 (5.19)	8 (5.41)	0.96	0.41–2.24	
**Anemia**					
No	305 (83.33)	135 (91.84)	Reference		0.013
Yes	61 (16.67)	12 (8.16)	2.25	1.17–4.32	
**Gestational diabetes**					
No	342 (93.96)	139 (93.92)	Reference		0.987
Yes	22 (6.04)	9 (6.08)	0.99	0.45–2.21	
**Thyroid disease**					
No	335 (91.78)	133 (89.86)	Reference		0.487
Yes	30 (8.22)	15 (10.14)	0.79	0.41–1.52	
**Smoker**					
No	291 (80.17)	116 (78.38)	Reference		0.649
Yes	72 (19.83)	32 (21.62)	0.90	0.56–1.43	
**Social drinker**					
No	195 (54.17)	84 (57.14)	Reference		0.541
Yes	165 (45.83)	63 (42.86)	1.13	0.77–1.66	

Cases and controls were asked to present any of these medical conditions from 6 months before pregnancy to delivery.

*OR*, odds ratio; *CI*, confidence interval.

*The number of cases varies because of missing data.

**Data from primiparous women were excluded for this analysis.

**Table 2:** Medical history of breastfed infants participating in the study

**Variables**	**Case, *n*** * (%)	**Control, *n*** * (%)	**OR**	**95% CI**	***P*-value**
**Sex**					
Female	157 (42.90)	78 (52.70)	Reference		
Male	209 (57.10)	70 (47.30)	1.48	1.01–2.18	0.043
**Blood type**					
A	104 (39.69)	51 (48.11)	Reference		
B	29 (11.07)	4 (3.77)	3.56	1.19–10.66	0.011
AB	16 (6.11)	1 (0.94)	7.85	1.01–60.82	
O	113 (43.13)	50 (47.17)	1.11	0.69–1.78	
**Rh factor**					
Positive	240 (91.60)	100 (94.34)	Reference		
Negative	22 (8.40)	6 (5.66)	1.53	0.60–3.88	0.370
**APGAR test**					
< 9	26 (7.60)	3 (2.26)	Reference		
> 9	316 (92.40)	130 (97.74)	0.28	0.08–0.94	0.049
**Jaundice**					
No	244 (67.22)	109 (74.15)	Reference		
Yes	119 (32.78)	38 (25.85)	1.40	0.91–2.15	0.125
**Hypoglycemia**					
No	357 (97.01)	143 (96.62)	Reference		
Yes	11 (2.99)	5 (3.38)	0.88	0.30–2.58	0.818
**Eczema**					
No	347 (94.29)	141 (95.27)	Reference		
Yes	21 (5.71)	7 (4.73)	1.22	0.51–2.93	0.658
**Thrush**					
No	328 (89.13)	143 (96.62)	Reference		
Yes	40 (10.87)	5 (3.38)	3.49	1.35–9.02	0.006
**Micrognathia**/**Retrognathia**					
No	355 (96.47)	147 (99.32)	Reference		
Yes	13 (3.53)	1 (0.68)	5.38	0.70–41.52	0.132
**Tongue**-**tie**					
No	255 (71.63)	134 (90.54)	Reference		
Yes	101 (28.37)	14 (9.46)	3.79	2.09–6.89	< 0.001
**Child hospitalized after birth**					
No	323 (90.73)	134 (96.40)	Reference		
Yes	33 (9.27)	5 (3.60)	2.74	1.05–7.16	0.033

*OR*, odds ratio; *C*I, confidence interval.

*The number of cases varies because of missing data.

**Table 3:** **Pregnancy**, **delivery and postpartum characteristics of participants in this study**

**Variables**	**Case, *n*** * (%)	**Control, *n*** * (%)	**OR**	**95% CI**	***P*-value**
**Threatened miscarriage**					
No	308 (85.08)	135 (91.22)	Reference		0.063
Yes	54 (14.92)	13 (8.78)	1.82	0.96–3.45	
**Breast**/**nipple pain during pregnancy**					
No	249 (70.94)	117 (80.14)	Reference		0.034
Yes	102 (29.06)	29 (19.86)	1.65	1.04–2.64	
**Antibiotics during pregnancy**					
No	271 (77.87)	122 (82.43)	Reference		0.252
Yes	77 (22.13)	26 (17.57)	1.33	0.81–2.18	
**Antifungal medication during pregnancy**					
No	317 (89.30)	138 (93.24)	Reference		0.170
Yes	38 (10.70)	10 (6.76)	1.65	0.80–3.41	
**Analgesics during pregnancy**					
No	233 (64.36)	105 (71.43)	Reference		0.126
Yes	129 (35.64)	42 (28.57)	1.38	0.91–2.10	
**Group B *Streptococcus *** **positive test**					
No	278 (79.89)	115 (80.99)	Reference		0.781
Yes	70 (20.11)	27 (19.01)	1.07	0.65–1.76	
**Age range at last delivery**					
< 25	5 (1.37)	5 (3.45)	Reference		0.305
25-35	242 (66.12)	95 (65.52)	2.55	0.72–9.00	
> 35	119 (32.51)	45 (31.03)	2.64	0.73–9.57	
**Primiparous**/**Multiparous**					
Multiparous	113 (30.87)	62 (42.18)	Reference		0.015
Primiparous	253 (69.13)	85 (57.82)	1.63	1.10–2.43	
**Place of delivery**					
Private clinic	118 (33.71)	25 (17.01)	Reference		0.021
Public hospital	224 (64.00)	113 (76.87)	0.42	0.26–0.68	
Home	8 (2.29)	9 (6.12)	0.19	0.07–0.54	
**Type of delivery**					
Vaginal	267 (76.95)	125 (85.03)	Reference		0.042
Caesarean section	80 (23.05)	22 (14.97)	1.70	1.01–2.86	
**Antibiotherapy during delivery**					
No	201 (57.59)	100 (67.57)	Reference		0.038
Yes	148 (42.41)	48 (32.43)	1.53	1.02–2.30	
**Epidural analgesia during delivery**					
No	73 (20.17)	48 (32.43)	Reference		0.003
Yes	289 (79.83)	100 (67.57)	1.90	1.24–2.92	
**First contact with child**					
Immediately	243 (66.21)	114 (77.51)	Reference		0.019
10-60 min	78 (21.25)	25 (17.01)	1.46	0.89–2.42	
> 60 min	46 (12.53)	8 (5.44)	2.70	1.23–5.90	
**Separation child**-**mother longer than 24 h**					
No	330 (89.92)	144 (97.30)	Reference		0.009
Yes	37 (10.08)	4 (2.70)	4.04	1.41–11.53	

*OR*, odds ratio; *CI*, confidence interval.

*The number of cases varies because of missing data.

**Table 4:** Breastfeeding characteristics and practices of the women involved in this study

**Variables**	**Case, *n*** * (%)	**Control, *n*** * (%)	**OR**	**95% CI**	***P*-value**
**First breastfeed after birth**					
Not immediately	104 (28.42)	24 (16.44)	Reference		0.005
Immediately	262 (71.58)	122 (83.56)	0.50	0.30–0.81	
**Problems to latch on the nipple at first**					
No	250 (67.93)	125 (85.03)	Reference		< 0.001
Yes	118 (32.07)	22 (14.97)	2.68	1.62–4.44	
**Time until the milk come in**					
Hours	50 (13.77)	42 (29.17)	Reference		< 0.001
One day	66 (18.18)	27 (18.75)	2.05	1.12–3.77	
Several days	247 (68.04)	75 (52.08)	2.77	1.70–4.49	
**Breast milk amount**					
Normal	185 (50.27)	94 (63.95)	Reference		0.006
Oversupply	139 (37.77)	48 (31.29)	1.54	1.01–2.33	
Low supply	44 (11.96)	7 (4.76)	3.19	1.39–7.36	
**Types of breastfeeding**					
Mixed	272 (75.35)	14 (9.52)	Reference		< 0.001
Exclusive	89 (24.65)	133 (90.48)	0.32	0.18–0.59	
**Breastfeeding twins/Tandem nursing**					
No	349 (96.41)	126 (87.50)	Reference		< 0.001
Yes	13 (3.59)	18 (12.50)	0.26	0.12–0.55	
**Breastfeeding positions**					
1	48 (13.30)	22 (15.28)	1.05	0.60–1.86	0.043
2	170 (47.09)	82 (56.94)	Reference		
3	143 (39.61)	40 (27.78)	1.72	1.11–2.67	
**Breastfeeding length**					
5-15 min	71 (26.59)	36 (41.38)	Reference		< 0.001
15-30 min	112 (41.95)	38 (43.68)	1.49	0.87–2.58	
30-45 min	37 (13.86)	8 (9.20)	2.35	0.99–5.56	
> 45 min	47 (17.60)	5 (5.75)	4.77	1.74–13.03	
**Time since breastfeeding started**					
< 2 weeks	31 (8.59)	3 (2.59)	Reference		< 0.001
2-4 weeks	61 (16.90)	8 (6.90)	0.74	0.18–2.98	
1-3 months	161 (44.60)	22 (18.97)	0.71	0.20–2.51	
3-6 months	52 (14.40)	51 (43.97)	0.10	0.03–0.34	
6-12 months	30 (8.31)	15 (12.93)	0.19	0.05–0.74	
> 12 months	26 (7.20)	17 (14.66)	0.15	0.04–0.56	
**Breast preference**					
No	233 (64.19)	104 (71.23)	Reference		0.129
Yes	130 (35.81)	42 (28.77)	1.38	0.91–2.10	
**Consecutive feeds start with same breast**					
No	331 (92.72)	137 (93.84)	Reference		0.655
Yes	26 (7.28)	9 (6.16)	1.20	0.55–2.62	
**One or two breast in each session**					
1	138 (43.13)	54 (40.30)	Reference		0.578
2	182 (56.88)	80 (59.70)	0.89	0.59–1.34	
**Child skips one breastfeed session**					
No	274 (96.14)	117 (99.15)	Reference		0.195
Yes	11 (3.86)	1 (0.85)	4.70	0.60–36.80	
**Pacifier**					
No	235 (66.38)	112 (75.68)	Reference		0.040
Yes	119 (33.62)	36 (24.32)	1.58	1.02–2.44	
**Nipple shields**					
No	291 (82.20)	141 (95.27)	Reference		< 0.001
Yes	63 (17.80)	7 (4.73)	4.36	1.95–9.77	
**Bottle**-**feeding**					
No	260 (73.45)	136 (91.89)	Reference		< 0.001
Yes	94 (26.55)	12 (8.11)	4.10	2.17–7.74	
**Flat or inverted nipples**					
No	323 (91.24)	147 (99.32)	Reference		0.002
Yes	31 (8.76)	1 (0.68)	14.11	1.91–104.34	
**Cream on nipples**					
No	171 (47.63)	108 (75.52)	Reference		< 0.001
Yes	188 (52.37)	35 (24.48)	3.39	2.20–5.24	
**Breast pumps**					
No	143 (39.29)	101 (69.18)	Reference		< 0.001
Yes	221 (60.71)	45 (30.82)	3.47	2.30–5.22	
**Breast pads**					
No	116 (32.77)	56 (38.89)	Reference		0.193
Yes	238 (67.23)	88 (61.11)	1.31	0.87–1.95	
**Brassier use**					
Day and night	170 (48.99)	72 (54.96)	Reference		0.244
Only day	177 (51.01)	59 (45.04)	1.27	0.85–1.90	
**Cracked nipples (1**–5)^**^					
1	73 (19.84)	77 (52.03)	Reference		< 0.001
2	42 (11.41)	26 (17.57)	1.70	0.95–3.06	
3	50 (13.59)	12 (8.11)	4.39	2.17–8.91	
4	43 (11.68)	9 (6.08)	5.04	2.30–11.07	
5	160 (43.48)	24 (16.22)	7.03	4.02–12.01	
**Antibiotics during breastfeeding**					
No	207 (56.71)	126 (85.71)	Reference		< 0.001
Yes	158 (43.29)	21 (14.29)	4.58	2.76–7.60	
**Antifungal medication during breastfeeding**					
No	280 (76.71)	132 (89.80)	Reference		< 0.001
Yes	85 (23.29)	15 (10.20)	2.67	1,49–4,80	
**Analgesics during breastfeeding**					
No	217 (58.97)	109 (73.65)	Reference		< 0.001
Yes	151 (41.03)	39 (26.35)	1.94	1.28–2.96	
**Non-steroidal anti-inflammatory drugs**					
No	218 (59.24)	119 (80.41)	Reference		< 0.001
Yes	150 (40.76)	29 (19.59)	2.82	1.79–4.45	
**Corticosteroids**					
No	355 (96.47)	146 (98.65)	Reference		0.297
Yes	13 (3.53)	2 (1.35)	2.67	0.60–11.99	

*OR*, odds ratio; *CI*, confidence interval.

*The number of cases varies because of missing data.

**Cracked nipples were rated from 1 (no cracked) to 5 (severely cracked).

### Statistical analysis

Continuous variables were expressed as means and 95% confidence intervals (95% CI) and percentages were calculated for categorical variables. Comparison of continuous and categorical variables in both control and mastitis groups was done using Student’s t-test and chi-squared test, respectively, and any statistically significant difference was noted. Fisher’s exact test was used as appropriate. Odds Ratio (OR) associated with each potential factor involved in mastitis (risk or protective factor) and 95% CI were calculated to compare exposures in each group. For the purpose of the study, *P* < 0.05 was considered statistically significant.

The relationship between mastitis risk and seventy-eight variables was first examined by bivariate analysis. To analyze the strength of association between mastitis and the potential risk factors, most factors that were statistically significant in the bivariate analysis, based on a *P* < 0.05 significance level (thirty-four variables), were used in a multivariate logistic regression model using the maximum likelihood method of the LOGISTIC procedure of SAS. Variables were included one at a time in the multivariate model using the forward stepwise procedure, adding the predictor with the largest score statistic. Variables which were significant by the Wald statistic at *P* < 0.05 were included in the final model. Adjusted OR (AOR) and 95% CI were calculated for the selected variables in the multivariate logistic regression model. Adequacy of the multivariate model was estimated by Hosmer-Lemeshow test and the area under the Receiver Operating Characteristic (ROC) curve.

The Statgraphics Centurion XVI software (16.1.03) (StatPoint Technologies, Inc., Warrenton, VA, USA) and the SAS software, version 9.2 (SAS Institute, Inc., Cary, NC, USA) were used for these analyses.

## Results

A total of 368 cases (mastitis) and 148 controls (healthy women) were included in the study after filling out a questionnaire designed to collect retrospective information about different factors that could be involved in mastitis.

Concerning demographic characteristics, there were no significant differences between case and control subjects with regard to mean age at last delivery (cases: 33.7 years, 95% CI: 33.01–34.19; controls: 33.6 years, 95% CI: 33.32–34.12; *P* = 0.756), infant weight (cases: 3.33 kg, 95% CI: 3.28–3.35; controls: 3.32 kg, 95% CI: 3.27–3.38; *P* = 0.786) and infant size (cases: 49.83 cm, 95% CI: 49.64–50.02; controls: 50.17 cm, 95% CI: 49.87–50.46; *P* = 0.186). However, the mean infant age when the mother completed the questionnaire was significantly different (*P* < 0.001) between cases (3.35 months; 95% CI: 2.91–3.79) and controls (6.68 months; 95% CI: 5.93–7.42).

The bivariate analysis for qualitative variables that could be related to mastitis is shown in Tables 1, 2, 3 and 4, where the results are presented as percentages, ORs and 95% CI.

The factors related to the medical history of the women participating in this study are shown in Table 1. Compared with controls, a history of mastitis in the family (OR: 3.20) or in previous lactations (OR: 7.13) were strongly associated with a higher risk of mastitis. There were no significant differences between the case patients and the control subjects with regard to blood type, Rh factor, history of breast cancer in the family, breast surgery or the presence of a gastrointestinal disease.

Women with mastitis were more likely to report urinary infection (OR: 1.77) and vaginal candidiasis (OR: 1.89) than controls, as well as throat (OR: 2.47) and skin infection (OR: 2.65). In addition, significantly more case-patients than controls reported anemia (OR: 2.25). No significant differences were found between both groups regarding smoking or drinking habit.

Some factors related to the infant medical history are shown in Table 2. Infant blood type B was significantly more reported by women with mastitis compared to controls (OR: 3.56). No differences were found related to Rh factor. However, there was a marginally significant difference regarding child gender, so that more case-patients declared to give birth to a male child compared to controls (OR: 1.48). APGAR test score > 9 was found to be a barely significant protective factor (OR: 0.28).

No significant differences were observed in relation to jaundice, hypoglycemia, eczema or micrognathia/retrognathia, but significantly more infants from mothers with mastitis suffered oral thrush (OR: 3.49). The presence of tongue-tie in the infant (OR: 3.79) and infant hospitalization after birth (OR: 2.74) were significantly more reported in the mastitis group.

Characteristics of pregnancy, delivery and postpartum are shown in Table 3. There was not a statistically significant difference between cases and controls related to a threatened miscarriage. In contrast, a history of breast/nipple pain during pregnancy was significantly more common among women with mastitis (OR: 1.65).

The use of antibiotics, antifungal treatment and analgesics during pregnancy between cases and controls was not statistically different. However, antibiotherapy (OR: 1.53) and epidural analgesia (OR: 1.90) during delivery were significantly more widely administered to women reporting mastitis. There were no age-related significant differences at delivery, but primiparous women were found significantly more often in the mastitis group (69.13%) than in the control group (57.82%).

Place and type of delivery also showed significant differences between cases and controls. Delivery in a public hospital (OR: 0.42) had a significant protective effect compared to delivery in a private clinic, while Caesarean sections were performed on women in the mastitis group at significantly higher frequency (23.05%) than on those in the control group (14.97%).

Contact with the infant immediately after birth was more likely reported by controls (77.51%) than cases (66.21%), while the mastitis group reported more often (12.53%) that the first contact took at least one hour after birth compared to controls (5.44%). A higher risk of mastitis was noted when the infant was separated from the mother for more than 24 h (OR: 4.04).

Breastfeeding characteristics and practices are shown in Table 4. Breastfeeding started immediately after birth significantly more often in controls than in cases (83.56% and 71.58%, respectively). Exclusive breastfeeding (OR: 0.32) and breastfeeding twins/tandem nursing (OR: 0.26) were also more common in the control group. Women having children with latching problems were about 2.7 times more likely to report mastitis (OR: 2.68), and similar results were found if there was a delay of several days in breast milk coming in (OR: 2.77). Perceiving a low milk supply (OR: 3.19) or an oversupply (OR: 1.54) also turned out to be described significantly most often by women suffering from mastitis.

Women with mastitis were more likely to use more breastfeeding positions (39.61%) than controls (27.78%) although it may be a consequence of the pain while breastfeeding. In addition, breastfeeding longer than 45 minutes was significantly associated with mastitis (OR: 4.77).

The use of pacifiers, bottle-feeding or nipple shields, that might interfere with proper suckling in some situations, was significantly more frequent among cases (approximately 1.6 times (OR: 1.58), 4.10 times (OR: 4.10) and 4.4 times (OR: 4.36) higher, respectively). The number of controls reporting flat or inverted nipples was insufficient to describe precisely the relationship between this condition and the risk of mastitis. Other breastfeeding practices strongly linked to women with mastitis included the use of creams (OR: 3.39) and pumps (OR: 3.47).

Women were asked to rate cracked nipples on a 5-point scale, from 1 (no cracked nipples) to 5 (severely cracked nipples). Cracked nipples rated 3 to 5 were more frequently reported by women with mastitis compared to controls, especially severely cracked ones (OR: 7.03).

Use of oral antibiotic (OR: 4.58) and topical antifungal (OR: 2.67) drugs was significantly associated with mastitis. Use of analgesics (OR: 1.94) and non-steroidal anti-inflammatory drugs (OR: 2.82) also showed significant differences between cases and controls. Oral corticosteroid therapies did not yield a significant difference since their use was very low among the analyzed sample.

All statistical significant variables related to mother medical history (Table 1) and pregnancy, delivery and postpartum characteristics (Table 3) were included in the multivariable logistic regression model. However, 8 variables with a *P* value < 0.05 were excluded from the multivariable analysis for several reasons. Regarding infant medical history (Table 2) the following were not included: sex and APGAR test (marginally significant difference), blood type (small sample size in controls), tongue tie (condition underrated in the control group) and child hospitalized after birth, because all of them were separated from their mother longer than 24 h and this factor was included in the multivariable analysis. Concerning breastfeeding characteristics and practices (Table 4) the variables not included were: breastfeeding positions (clearly linked to pain in women with mastitis), flat or inverted nipples (small sample size in controls) and breastfeeding length.

AOR, 95% CI, and *P* values of the multivariate logistic-regression model determined by forward stepwise selection are shown in Table 5. After adjustment for potentially correlated covariates, the factors significantly- and independently-associated with mastitis were history of mastitis in the family (AOR: 2.28), mastitis in previous lactations (AOR: 3.91) and throat infection (AOR: 2.05), in relation to the history of the mother. A mother-infant separation longer than 24 h after birth increased the risk of suffering mastitis about 6 times (AOR: 6.40). Regarding breastfeeding, the variables most significantly- and independently-associated with mastitis were infant age (AOR: 0.92), breast milk coming in later than 24 h postpartum (AOR: 2.26), cracked nipples (AOR: 1.43) and use of creams (AOR: 1.91), breast pumps (AOR: 2.78), oral antibiotics (AOR: 5.38) and topical antifungal medication (AOR: 3.81).

**Table 5:** Risk factors for mastitis according to multiple logistic regression analysis

**Variables**	**Adjusted OR**	**95% CI**	***P*-value**
Cracked nipples	1.43	1.23–1.67	< 0.0001
Antibiotics during breastfeeding	5.38	2.85–10.14	< 0.0001
Infant age	0.92	0.87–0.96	< 0.0001
Breast pumps	2.78	1.68–4.58	< 0.0001
Antifungal medication during breastfeeding	3.81	1.35–10.76	0.0009
Mastitis in previous breastfeeding	3.91	1.60–9.56	0.0014
Breast milk coming in later than 24 h	2.26	1.24–4.12	0.0016
Mastitis in the family	2.28	1.26–4.13	0.0028
Separation child-mother longer than 24 h	6.40	1.77–23.18	0.0027
Cream on nipples	1.91	1.13–3.24	0.0228
Throat infection	2.05	1.10–3.80	0.0224

*OR*, odds ratio; *CI*, confidence interval.

The following variables have been included in the analysis: infant age; mastitis in the family and in previous breastfeeding; urinary, throat and skin infection; vaginal candidiasis; anemia; thrush; breast/nipple pain during pregnancy; parity; place and type of delivery; antibiotherapy and analgesia during delivery; first contact with child; separation child-mother longer than 24 h; first breastfeed after birth; problems to latch on nipple at first; time until the milk comes in; milk amount; mixed/exclusive breastfeeding; breastfeeding two children; time since breastfeeding started; pacifier; nipple shields; bottle-feeding; cream on nipples; breast pumps; cracked nipples; antibiotics, antifungal medication, analgesics and non-steroidal anti-inflammatory drugs during breastfeeding.

The Hosmer-Lemeshow goodness-of-fit test showed a Chi-square of 7.32 (*P* = 0.503) and the area under the ROC curve was 0.870 (95% CI = 0.835-0.904), which means that the model presented a good fit and a good adjustment.

## Discussion

The purpose of this case–control investigation was to identify factors associated with mastitis, including potential risk or protective factors. Among them, the separation of the infant from his mother longer than 24 h after birth due to hospitalization or for any other reason increased the risk of mastitis. This highlights the crucial importance of the first postnatal hours for establishing mother-infant interaction. Among other aspects of pregnancy, delivery and postpartum, Caesarean delivery and antibiotherapy during delivery as well as the use of epidural analgesia in labor were more frequently reported by mothers with mastitis, although these factors were not included in the final model obtained after the multivariable analysis. In this sense, a negative association between Caesarean delivery and breastfeeding exists because postoperative care routines delay the onset of lactation, disrupt mother-infant interaction and inhibit infant suckling [20]. Peripartum antibiotherapy has emerged as a strong risk factor for human mastitis because it induces the selection for antibiotic-resistant bacteria in the mammary gland and the elimination of potential competitors [21,22]. Antibiotics also affect vaginal and intestinal microbiota of the mother [22] and the development of intestinal microbiota in the infant [23]. The link between epidural intrapartum analgesia and breastfeeding difficulties has also been debated [24-26], but there are not conclusive evidences and further studies are required.

Another relevant factor associated with mastitis was the use of antibiotics during breastfeeding. In fact, widespread use of broad spectrum antibiotics is leading to increasing rates of antimicrobial resistance among mastitis-causing agents [27-29]. On the other hand, biofilm formation is an important virulence factor of the strains implicated in mastitis, taking in account that the penetration capacity through bacterial biofilms depends on each antibiotic [30]. Resistance to antibiotics and ability to form biofilms are common findings among mastitis-causing strains and may explain the often recurrent nature of this infectious condition [21]. This fact emphasizes the need of a milk culture and antibiogram for a rational treatment of mastitis [19,31]. Widespread antibiotic therapy used to treat throat infections could also affect the mammary gland microbiota and lead to mastitis. Conversely, broad range antibiotics to treat mastitis are linked to a variety of adverse effects, including urinary infections and vaginal candidiasis [32]. Microbial habitats in the human body, including the skin, constitute a network of interrelated communities [33]. This could explain why pathogens involved in throat and urinary infections may spread to the mammary gland and those implicated in mastitis development may spread to throat and urinary tract. In this work, the bivariate analysis revealed that urinary and skin infections were also more frequent among breastfeeding mothers with mastitis. Interestingly, anemia was also more common in the group of women with mastitis, since women suffering from anemia might be more vulnerable to infections. In addition, iron supplements enhance growth and virulence of *Staphylococcus aureus* and other mastitis-associated species [34]; as a consequence, women receiving them may also be more prone to mastitis. No clinical trials have evaluated the impact of iron supplementation on mastitis but the study of iron acquisition pathways seems to be a good target to define the underlying mechanisms of mastitis severity [35].

Significantly, a history of mastitis with a previous infant also seems to be a strong mastitis predictor [1,14] and, in our study, this factor was associated with an almost four-fold risk of mastitis in the multivariate analysis. Breast health depends on a balanced interaction between the host and its microbiota [36,37]. Since the milk bacterial profile is host-specific [36,38,39], there could be a mammary microbiota more prone to mastitis development [6]. In fact, *S. epidermidis*, an underrated cause of mastitis, lives at the edge between commensalism and pathogenicity and requires a predisposed host to transform itself into a notorious pathogen [40,41]. On the other hand, several oligosaccharides involved in the mucosal immune system are present in human milk [42]. Therefore, differences in profile and concentration of such compounds may explain a differential host susceptibility to develop mastitis [43].

Our results indicate that a familial occurrence of mastitis is a significant risk factor for the disease, which suggests the role of a genetic predisposition in the development of mastitis. The existence of a genetic basis for host responses to bacteria involved in mastitis has been widely documented in cattle and sheep mastitis [44,45], although the underlying mechanisms are still largely unknown. The first association between human granulomatous mastitis caused by *Corynebacterium kroppenstedtii* and a single nucleotide polymorphism (SNP) related to defective neutrophil responses has been recently described [46], which opens new fields for further investigation in human mastitis.

In our work, antifungal medication during breastfeeding was strongly linked to mastitis. Antifungal ointments are often prescribed to treat “mammary candidiasis” on the basis of visual assessment, without a microbiological analysis. Actually, yeasts are an extremely rare cause of lactational mastitis in any mammalian species and there is lack of evidence to reach such a diagnosis [47-49]. It is interesting to note, however, that there is an association between staphylococcal/streptococcal mastitis and candidiasis (oral thrush) in the infant since a high concentration of such bacteria can induce *Candida albicans* overgrowth. *C. albicans* and streptococci form a synergistic partnership where *Streptococcus* promotes fungal growth by secreting metabolic products that can be used as a carbon source by the yeast [50,51]. After *C. albicans* overgrowth in the infant mouth, some of the yeast cells can be transferred to the mother through breastfeeding, so that *C. albicans* could be isolated from breast milk and misdiagnosed as the cause of mastitis.

On the other hand, nipple cracks have been significantly associated with mastitis in previous studies [1,7,13-15] under the hypothesis that it provides a portal of entry for microorganisms. However, recent studies suggested that nipple lesions can be a precocious clinical sign of mastitis rather than a predisposing factor [21]. Exfoliative or “epidermolytic” toxins are relevant virulence factors of *S. aureus* and other *Staphylococcus* species [52]. In fact, an increased milk concentration of staphylococci or streptococci had increased odds for damaged nipples [53]. Our results also revealed that the use of ointments was associated with increased incidence of mastitis, in agreement with previous studies [1,14]. Such practice may provide good environmental conditions for bacterial overgrowth and dissemination.

The use of breast pumps was associated with mastitis, although this fact may be a consequence rather than a cause, since pumping is frequently recommended to reduce breast pressure and diminish bacterial load inside the mammary ducts during mastitis [14]. Too much expression may also result in pain from breast overstretching while improper use of an electric pump can lead to mastitis, trauma, and nipple wounds [1,54].

Regarding breastfeeding characteristics and practices, the risk of developing lactational mastitis was also associated with the breast milk coming in later than 24 h postpartum. Previous studies focused on a potential relationship between positioning, attachment problems and mastitis have provided contradictory results [13,55]. The bivariate analysis showed that mastitis was less common in women that breastfed her infant immediately after birth and also in women whose infants did not have difficulties in the first latch. In fact, it has been reported that timing of the first feeding is a key determinant for establishing mother-infant interaction and breastfeeding success [56]. Other factors more frequently found in control women were feeding two children and exclusive breastfeeding *versus* mixed feeding while the opposite was detected for bottle-feeding, in agreement with other studies [1]. Exclusive breastfeeding not only avoids the use of nursing bottles, but provides better interaction between mother and infant; therefore, increasing the nursing frequency and contributing to an adequate milk drainage. Considering these facts, breastfeeding twins or tandem nursing might be also considered a protective factor for mastitis. Regarding the amount of milk produced, milk over- or undersupply *versus* normal supply was more likely reported by cases. It has been suggested that mastitis may arise from a higher milk supply because of the risk of milk stasis if the infant delays or misses feeds [13]; this situation may provide good conditions for bacterial overgrowth. On the other hand, low milk supply could give to the mother a false perception of low milk production when, actually, only secretion is compromised due to the formation of thick bacterial biofilms inside the milk ducts [6,22,57]. This situation may also lead to longer feedings that were reported by women with mastitis.

Traditionally, interferences with suckling (pacifiers, bottle-feeding or nipple shields) have been related to breastfeeding problems and their use should be avoided, at least while the infant is learning to suckle properly. In our study, those practices were reported more frequently by women with mastitis when the initial bivariate analysis of possible risk factors was performed. In this analysis, a higher prevalence of tongue-tie (ankyloglossia) was found among infants from women with mastitis. However, this condition is not usually considered in infants without breastfeeding difficulties and, consequently, it could be underrated in the control group. The relationship between ankyloglossia and breastfeeding problems has been largely debated. Controversies in this area have arisen from attempts to find an absolute relationship between tongue-tie and breastfeeding difficulties instead of a relative one, where the first increases the risk for the latter [58]. Also, the existence of various classification systems for the diagnosis of ankyloglossia is confusing for clinicians.

Finally, mastitis was apparently associated with the lactation period, and the risk of mastitis decreased with the infant age (OR = 0.92). This could be a confounding factor because there was an age difference between cases (mean 3.35 months) and controls (mean 6.68 months) in spite of the fact that the same midwives and lactation consultants attending various health centers were in charge of recruiting cases (mastitis) and controls (healthy) women over a 21-month period. Actually, this difference in infant age due to unrestricted sampling of subjects may reflect the circumstance that lactational mastitis develops more frequently in the early stages of lactation. A higher incidence of mastitis during the first 4 weeks of breastfeeding and 75–95% of cases observed in the first 3 months has been reported previously [6]. A reduced number of the variables considered in the study could be influenced by infant’s age, including jaundice, hypoglycemia or eczema symptoms; however, the frequency of these conditions did not differ between cases and controls in the initial bivariate analysis. Regarding the length of feedings, feedings longer than 45 min were more frequently reported by cases. This fact could be related with younger children in cases compared to controls since the younger is the child, the longer is the feeding, but it might also be linked to a subclinical mastitis characterized by a decreased milk secretion that induces the baby to have longer feedings. However, taking these facts into account, this variable (feeding longer than 45 min) was excluded from the multivariable analysis and further studies will be carried out to clarify the link between breastfeeding length and mastitis.

We must acknowledge that there are other limitations in this study. Firstly, women had a strong commitment to breastfeeding since many of them were members of breastfeeding support groups. Secondly, the data were obtained retrospectively, which leads to a reporting bias due to lack of information about some questions. And thirdly, the results must be carefully interpreted because some identified associations could be consequences of mastitis rather than its causes, which is also a limitation in the design of case–control studies. On the other hand, further studies are needed to confirm the relationship between mastitis and all the exploratory variables of this study that have not been considered in previous research findings. Additionally, the long and complex questionnaire, as well as the elapsed time from the analyses of milk samples to the questionnaire reception, accounted for the low response rate in cases (34%). In fact, this rate would have been improved if the questionnaire had been delivered and responded to by the time the breast milk samples were collected to be analyzed. This fact will be taken into account in future studies to increase the response rates.

## Conclusions

Relevant factors related to an increased risk of infectious mastitis have been identified in this study. This knowledge will allow practitioners to provide appropriate management advice about modifiable risk factors, such as the use of pumps or inappropriate medication. They also could identify those women at an increased risk of developing mastitis, such as those having a familial history of mastitis, before delivery, and thus develop strategies to prevent this condition. There are still many questions to answer about infectious mastitis, but work is in progress to broaden our knowledge in this relevant Public Health issue.

## Competing interests

The authors declare that they have no competing interests.

## Authors’ contributions

PM assisted with the study design and data collection. LM contributed to analysis and interpretation of data. JMR originated and directed the study. LF and JMR reviewed the manuscript. MM conducted the study design, analyses and interpretation of the data and wrote the article. All authors read and approved the final manuscript.

## Pre-publication history

The pre-publication history for this paper can be accessed here:

http://www.biomedcentral.com/1471-2393/14/195/prepub
